# Developing a tool to assess motivation among health service providers working with public health system in India

**DOI:** 10.1186/s12960-016-0111-1

**Published:** 2016-04-14

**Authors:** Bhaskar Purohit, Abhishek Maneskar, Deepak Saxena

**Affiliations:** Indian Institute of Public Health Gandhinagar (PHFI), Drive in Road, Ahmedabad, 380054 India; Aarti Dental Clinic, Vigyan Nagar, Indore, 452012 India

**Keywords:** Motivation, Health service providers, India

## Abstract

**Background:**

Addressing the shortage of health service providers (doctors and nurses) in rural health centres remains a huge challenge. The lack of motivation of health service providers to serve in rural areas is one of the major reasons for such shortage. While many studies have aimed at analysing the reasons for low motivation, hardly any studies in India have focused on developing valid and reliable tools to measure motivation among health service providers. Hence, the objective of the study was to test and develop a valid and reliable instrument to assess the motivation of health service providers working with the public health system in India and the extent to which the motivation factors included in the study motivate health service providers to perform better at work.

**Methods:**

The present study adapted an already developed tool on motivation. The reliability and validity of the tool were established using different methods. The first stage of the tool development involved content development and assessment where, after a detailed literature review, a predeveloped tool with 19 items was adapted. However, in light of the literature review and pilot test, the same tool was modified to suit the local context by adding 7 additional items so that the final modified tool comprised of 26 items. A correlation matrix was applied to check the pattern of relationships among the items. The total sample size for the study was 154 health service providers from one Western state in India. To understand the sampling adequacy, the Kaiser-Meyer-Olkin measure of sampling adequacy and Bartlett’s test of sphericity were applied and finally factor analysis was carried out to calculate the eigenvalues and to understand the relative impact of factors affecting motivation.

**Results:**

A correlation matrix value of 0.017 was obtained narrating multi-co-linearity among the observations. Based on initial factor analysis, 8 out of 26 study factors were excluded from the study components with a cutoff range of less than 0.6. Running the factor analysis again suggested the inclusion of 18 items which were subsequently labelled under the following heads: transparency, goals, security, convenience, benefits, encouragement, adequacy of earnings and further growth and power.

**Conclusions:**

There is a great need to develop instruments aimed at assessing the motivation of health service providers. The instrument used in the study has good psychometric properties and may serve as a useful tool to assess motivation among healthcare providers.

## Background

Although the health workforce is arguably one of the most critical components of the health system and has a strong impact on overall health system performance [1], there is a worldwide estimated shortage of 4.3 million health workers with as many as 57 countries with severe shortage of health workers [2]. Inadequate number of healthcare workers is associated with poor quality of health services, especially in rural areas [3]. Therefore, an effective healthcare system needs to have an adequate-sized, well-motivated and skilled healthcare workforce [4].

The Indian public healthcare system suffers with severe shortage of workforce [5]. Such shortage is particularly evident in rural areas. The overall country figures for India suggest that the vacancy rate of medical officers (MOs) is nearly 21 % at primary health centres (PHCs) and 42 % for specialists at community health centres (CHCs) [6]. This problem is further aggravated with low levels of healthcare providers’ motivation. Lack of motivation has often been identified as a major problem in human resource crisis and, consequently, health service delivery and quality [7]. India’s existing disease burden and the changing demographic and disease profile warrant immediate attention to addressing the numeric inadequacy of health workers in order to achieve even modest coverage for essential health interventions [8]. However, the numeric inadequacy cannot be completely addressed unless the motivation of existing healthcare workers to improve the performance of the healthcare system is thoroughly understood and addressed. Assessing motivation is also very important because it is one of the most important factors for employees to perform better at work and to increase the productivity of an organization [9].

While there are many studies that have aimed at assessing motivation among healthcare providers in India [10–14], there is a dearth of studies done in India that have aimed at developing tools to assess motivation among health service providers working with the public health system in India. However, several studies conducted outside India have not only aimed at assessing motivation and job satisfaction among health service providers but also comment on the psychometric properties of the tools used to assess motivation and job satisfaction [15–19]. Hence, the aim of the current study was to test and develop a reliable and valid instrument for investigating the motivation of health service providers towards certain job-related aspects and the extent to which these motivate them to perform better at work.

### Theories of motivation relevant to study

While there are many theories of motivation, we only discuss two theories of motivation, Herzberg’s two-factor theory of motivation and Maslow’s need hierarchy theory of motivation, as the motivational factors included in the study fit closely to these theories [17]. Further, Maslow’s and Herzberg’s theories are relevant to public healthcare settings in India and the literature published on motivation in India covers many factors that have been proposed by Herzberg [14]. According to Herzberg’s two-factor theory of motivation, the factors that cause job satisfaction at work (which Herzberg calls motivators/intrinsic factors/job content factors) are different from the ones that cause job dissatisfaction if not met or prevent dissatisfaction if met (which he calls hygiene/extrinsic/job context factors). An example of motivators or intrinsic factor is recognition which, if met in a job, produces positive job satisfaction. On the other hand, hygiene/extrinsic factors, such as high salary, if not met, produce job dissatisfaction. According to this theory, the factors causing satisfaction are different from those causing dissatisfaction; hence, the two feelings should not be treated as opposites of one another [20–22].

Similarly, according to Maslow’s need hierarchy theory, employees have five levels of needs that can be explained with the help of a five-level pyramid. The lowest on the pyramid are physiological needs or basic needs such as salary and work conditions. The next level needs are safety needs such as safe working environment, insurance and job security. Next come the social or love needs like supportive team workers. The fourth level needs in the pyramid are self-esteem or ego needs such as status, responsibilities and recognition. And finally, on top the pyramid are self-actualization needs such as job challenges and creativity [23]. It is important to observe that there is a relationship between the Maslow need hierarchy theory and the two-factor theory of Herzberg. The lower level needs in the pyramid of Maslow’s theory, i.e. physiological needs, safety needs and social needs, correspond to the hygiene factors proposed by Herzberg, and the top two level needs in Maslow’s need hierarchy theory correspond to motivators or intrinsic factors.

## Methods

### Study area

The study was conducted in two districts from the state of Madhya Pradesh (MP) located in the Western part of India. The literature review for the study was done from December 2012 to February 2013 while the data collection was done from April to June 2013. A total of six blocks, three from each of the two randomly selected districts, were included in the present study. The study tried to include all the available doctors or medical officers (MOs) and nurses, both auxiliary nurse midwives (ANMs) and general nurse midwives (GNMs), from the selected six blocks. However, the state of MP suffers from critical shortage of health service providers, most notably the MOs and specialists working with rural health centres, i.e. PHCs and CHCs. For example, the state of MP has an overall vacancy rate of 53 % for MOs working with PHCs while the vacancy rate for MOs working as specialists with CHCs is around 51 % [6].

### Study population and sampling design

Efforts were done to include MOs, ANMs and GNMs from the six blocks within two selected districts working with PHCs, CHCs and district hospitals (DHs). Data was collected by visiting the health centres. All the MOs, ANMs and GNMs available at the time of data collection and those who were willing to participate in the study were included. None of the approached healthcare providers available at the time of data collection refused to participate in the study. However, due to overall shortage and absenteeism of healthcare providers, a total of only 154 respondents were included in the study.

### Tool development

To build a solid theoretical underpinning on motivation and to understand the motivation factors important among health workers, a detailed review was done focusing on Indian and international literature. The in-depth review included search on available literature in the form of published articles on motivation and job satisfaction from India and elsewhere. This process led to the inclusion of several papers from India [10–14] and elsewhere [24–32] and the inclusion of several important textbooks on organizational behaviour that touch upon theories of motivation [9, 33–35]. The literature review focused specifically on India in the form of papers published suggested several motivational factors important for healthcare workers’ will to perform better at work [10–14]. Attempts were later made to weave these factors into motivational theories that were most pertinent to the current research. Our literature review suggested two theories of motivation that were found most pertinent in this regard: Herzberg’s two-factor theory of motivation and Maslow’s need hierarchy theory as discussed above [12–15].

The next step was to identify instruments/tools that captured major motivation or job satisfaction factors. This literature review specifically focused on the identification of instrument/tools to explore how the main motivation factors identified earlier knit into the two motivational theories. Attempts were made to only include those instruments with psychometric properties such as established reliability and construct validity. This resulted in the identification of several papers from outside India [15–19, 36–40] but only two from India [41, 42]. From the review on motivational theories and papers on assessing motivation with established psychometric property review, we identified constructs that we thought were most appropriate to assess motivation. As described above, this led to the inclusion of several papers [15–19, 36–42] that measured different constructs or motivation. In order to establish content validity, two experts working on issues of motivation were involved throughout the stage of tool development. These experts were later involved to identify the most relevant constructs for the current study.

One of the instruments that most closely represented the motivational factors identified during literature review was the one used in a study conducted in Cyprus with health workers from a general hospital [43]. Another tool developed and tested for its psychometric properties from Kenya was considered for the study [16]. Although the tool used in Kenya was useful, it did not reflect several motivation factors from the theories of motivation that form the underpinning for our current research. The instrument from Cyprus better represents the factors based on motivation theories that form the basis for our research. However, a few of the motivation factors included in the Kenya study were already reflected in the Cyprus study. Hence, the Cyprus tool was more suited for the study. The Cyprus tool was adapted for the current study, however, with certain modifications discussed later in the “Methods” section. The authors of the study felt that a few motivational items included in the Cyprus study were not very explicit; hence, one of the modifications was to make these items more explicit in the study to avoid any ambiguity from the respondent’s side to understand the items. The Cyprus tool contained 19 items which are grouped under 4 different motivational factors, namely the following: *job attributes*, *remuneration*, *co-workers* and *achievement. Job attributes* included the following: authority, goals, creativity, clear duty, job control, skill exploitation and decision-making. *Remuneration* included the following: salary, work environment, retirement and absenteeism. *Co-workers* included the following: team work, job-related pride, appreciation, supervisor and fairness. *Achievement* included the following: meaningfulness, respect and interpersonal relationship [43].

However, to fit the instrument into the Indian health system context, the instrument was further modified to incorporate a few additional motivational factors relevant to the Indian healthcare system as presented in various published literature [9, 12, 14]. The inclusion of additional items was based on literature review specific to India that indicated the need to include items such as job security, challenging work, interesting work, growth and development in the adapted tool from the Cyprus study. During the literature review phase, the authors found two papers [17] that were very comprehensive that not only included nearly all items (from the Cyprus study and items relevant for the Indian context) but also contained some additional items that the authors felt were important representing the two motivational theories discussed above. So the adapted tool on motivation that contained 19 items was further modified to include 7 additional subfactors which were as follows: job security, availability of adequate resources, physical safety, challenging and interesting work, freely expressing opinion, and achievement-related promotion and growth and development. Hence, the final study instrument had a total of 26 subfactors. Responses were provided on a five-point unipolar scale corresponding to a five-point Likert’s scale, in which 1 corresponded to “not at all”, 2 to “a little bit”, 3 to “moderately”, 4 to “very” and 5 to “extremely”. These statements measured how important each subfactor of motivation was for increasing the respondents’ will to perform better at work with higher scores indicating higher motivation and vice versa.

The additional seven subfactors were added after a consensus-developing process among the two experts working on issues of motivation. A two-stage Delphi technique was used to build up the consensus between the experts and the co-authors [44]. These factors were also added as they were found very relevant to the Indian public health context [9, 12, 14].

#### Pilot testing

The pilot testing of the tool was done with 14 MOs and 5 nurses working with government health centres from Gujarat (another state), India, during February 2013 in order to get insight into the constructs selected for the study. The data collected during the pilot indicated that the tool was easy to understand and fill by the health service providers. The final instrument comprised of two sections. The first section contained questions on demographic- and job-related factors such as gender, place of work, type of service contract and years of experience. The second part contained 26 questions on intrinsic and extrinsic factors of motivation based on Maslow’s and Herzberg’s theories of motivation. The questions in the instrument were jumbled in a way that respondents did not have any clue of what extrinsic and intrinsic factors were.

### Data analysis

Validity for the instrument was established during the instrument development stage. Content validity was established by consulting two subject experts and by doing an expensive literature review as explained above. In order to check the tool’s reliability, the Cronbach alpha test was carried out. To establish construct validity [16, 45, 46], we calculated average variance and correlation scores. These scores were used to calculate the two subtypes of construct validity: convergent validity [47] and discriminant validity [48]. To ensure sampling adequacy, the Kaiser-Meyer-Olkin measure of sampling adequacy and Bartlett’s test of sphericity were also applied. Finally, factor analysis was conducted before and after extraction of common variance to calculate the eigenvalues. The data was analysed using SPSS version 19 [49].

### Research ethics

Informed written consent of the participants was taken before data collection. The participation in this study was voluntary, and the study assured to maintain complete anonymity of the study participants at all times. Necessary permission for the study was also taken from appropriate district authorities. The ethical approval for the study was obtained from the institutional ethical review committee at the Indian Institute of Public Health Gandhinagar (IIPHG).

## Results

A total of 154 participants were included in the study. Out of the total 154, 30 % were MOs, 46 % were ANMs and 24 % were GNMs. A high female participation of 72 % was observed in the study against 28 % of male respondents. Majority of the participants (around 60 %) were from PHCs while 22 % and 18 % were from CHCs and DHs, respectively. A total of around 86 % of respondents were on regular posting while the rest (14 %) had either ad hoc or bonded appointment. See Table 1 for details.

**Table 1:** Distribution of study respondents based on work related profile

Category	Number	Percent
MO	46	29.87
ANM	71	46.10
GNM	37	24.03
Total	154	100.00
Gender	Number	
Male	43	27.92
Female	111	72.08
Total	154	100.00
Place of work	Number	
DH	28	18.18
CHC	33	21.43
PHC	93	60.39
Total	154	100.00
Type of service	Number	
Bonded	22	14.29
Regular	132	85.71
Total	154	100.00
Years of service	Number	
Less than 2 years	25	16.23
2–5 years	31	20.13
More than 5 years	98	63.64
Total	154	100.00

A correlation matrix was applied to check the pattern of relationships among the factors included in the study. A correlation matrix value of 0.017 was obtained, which was greater than the necessary value of 0.00001 narrating multi-co-linearity among the observations noticed.

Next, the Kaiser-Meyer-Olkin (KMO) measure and Bartlett’s test of sphericity for sampling adequacy were calculated. As a convention, KMO statistics ranges between 0 and 1 and values greater than 0.5 are considered acceptable [50]. The observations in the present data had a KMO value of 0.541 reflecting a moderate acceptance value. On application of Bartlett’s test for null hypothesis, a significant value of 0.000 was obtained, suggesting that the original correlation matrix to be an identity matrix. (See Table 2 for details.)

**Table 2:** KMO and Bartlett’s test

KMO measure of sampling adequacy	.541
(Approx. chi square)	590.73
Bartlett’s test of sphericity (df)	351
Significance	.000

In order to check the tool’s reliability, the Cronbach alpha test statistic was calculated by taking all the questions together as a single index of motivation that suggested the Cronbach alpha test statistic value of 0.81, an acceptable value for the tool.

The extent to which the motivation factors included in the study motivate health service providers to perform better at work is provided in Table 3. The results suggest that under the job attribution heading, availability of adequate resources was found to be the most important motivation factor for all the three categories of respondents, i.e. MOs, ANMs and GNMs. Similarly, under the remuneration heading, good working environment was found to be the most important motivation factor. Under the co-worker heading, supervisors’ support was found to be the most important, while under achievement heading, achievement-related promotion was reported to be the most important by all the three categories of health workers included in the study.

**Table 3:** Mean score and (SD) of 26 motivation factors by type of health service provider

Factors under job attributions		Mean score for MO	Mean score for ANM	Mean score for GNM
1	Exercising authority	4.83 (0.46)	4.76 (0.46)	4.92 (0.46)
2	Significant and meaningful goal	4.09 (0.31)	4.07 (0.31)	4.14 (0.31)
3	Creative opportunity	4.11 (0.51)	4.20 (0.51)	4.19 (0.51)
4	Clear duties and responsibility	4.78 (0.37)	4.92 (0.37)	4.81 (0.37)
5	Control over job decision related to utilizing money	4.17 (0.57)	4.15 (0.57) 4.27 (0.57)	
6	Job security	4.59 (0.57)	4.49 (0.57)	4.62 (0.57)
7	Opportunity to use Skills	4.22 (0.62)	4.04 (0.62)	4.24 (0.62)
*8*	*Availability of adequate resources*	*4.93 (0.29)*	*4.93 (0.29)*	*4.89 (0.29)*
9	Physical safety	4.74 (0.40)	4.87 (0.40)	4.89 (0.40)
10	Challenging and interesting work	4.43 (0.51)	4.44 (0.51)	4.51 (0.51)
11	General decision-making	3.85 (0.79)	4.03 (0.79)	4.05 (0.79)
Factors under remuneration				
12	Adequate salary and benefits	4.48 (0.61)	4.62 (0.61)	4.86 (0.61)
13	Pension	3.78 (0.64)	4.30 (0.64)	4.35 (0.64)
*14*	*Good working environment*	*4.65 (0.49)*	*4.68 (0.49)*	*4.89 (0.49)*
15	Adequate leaves	3.72 (0.62)	3.79 (0.62)	3.89 (0.62)
Factors under co-worker				
16	Effective team work	4.85 (0.34)	4.83 (0.34)	4.95 (0.34)
17	Job-related pride and respect	4.20 (0.46)	4.27 (0.46)	4.16 (0.46)
18	Freely expressing opinion	4.13 (0.48)	4.23 (0.48)	4.16 (0.48)
19	Appreciation for good work	4.26 (0.61)	4.25 (0.61)	4.19 (0.61)
*20*	*Supervisor’s support*	*4.91 (0.31)*	*4.89 (0.31)*	*4.95 (0.31)*
21	Fair treatment by colleagues	3.93 (0.46)	4.14 (0.46)	4.14 (0.46)
Factors under achievements				
22	Job meaningfulness	4.11 (0.47)	4.21 (0.47)	4.24 (0.47)
23	Earned respect as a person	4.09 (0.54)	4.14 (0.54)	4.30 (0.54)
*24*	*Achievement-related promotion*	*4.65 (0.52)*	*4.69 (0.52)*	*4.62 (0.52)*
25	Growth and development	4.43 (0.54)	4.23 (0.54)	4.32 (0.54)
26	Interpersonal relationship	3.89 (0.60)	4.18 (0.60)	3.78 (0.60)

As a next step, factor analysis was carried out. Table 4 describes the factorial analysis of communalities before and after extraction of individual factors. Principal component analysis works on the initial assumption that variance should be common before extraction communities and should be equal to 1. The extraction values reflect the common variance in data structure. However, after extraction, 8 of the total 26 items/subfactors with values less than 0.6 were discarded as values less that 0.6 indicate variables that do not fit well with the factor solution. As explained in the table below, 8 of the total 26 items that were excluded from further analysis as their values were less than 0.6 were as follows: creative opportunities, opportunity to use skill acquired through professional course, general decision-making, availability of adequate resources, job security, freely expressing opinion, fair treatment by colleagues and lastly challenging and interesting work. (See Table 4 for details.)

**Table 4:** Distribution of the statements narrating the factors associated with motivation after extraction

Statements/items	Initial	Extraction	Rotated component matrix
1. Exercising authority	1.000	.643	Included
2. Significant and meaningful goal	1.000	.732	Included
3. Creative opportunities	1.000	.593	Excluded
4. Clear duties and responsibilities	1.000	.608	Included
5. Control over job decision related to utilizing money, procurement, HR	1.000	.675	Included
6. Opportunity to use skill acquired through professional course	1.000	.588	Excluded
7. General decision-making (day to day affairs)	1.000	.560	Excluded
8. Availability of adequate resources (money)	1.000	.544	Excluded
9. Adequate salary and benefits	1.000	.686	Included
10. Pension	1.000	.636	Included
11. Good working environment	1.000	.646	Included
12. Adequate leaves	1.000	.709	Included
13. Job security	1.000	.550	Excluded
14. Achievement-related promotion	1.000	.602	Included
15. Freely expressing opinion	1.000	.542	Excluded
16. Effective team work	1.000	.652	Included
17. Job-related pride and respect	1.000	.656	Included
18. Appreciation for good work	1.000	.613	Included
19. Supervisor’s support	1.000	.784	Included
20. Fair treatment by colleagues	1.000	.559	Excluded
21. Growth and development	1.000	.678	Included
22. Job meaningfulness	1.000	.666	Included
23. Earned respect as a person	1.000	.641	Included
24. Interpersonal relationship	1.000	.605	Included
25. Physical safety	1.000	.747	Included
26. Challenging and interesting work	1.000	.522	Excluded

For establishing construct validity, we conducted the pattern matrix analysis wherein average loading of each factors (F-1 to F-8) were calculated. Then from average loading, we extracted the variance, i.e. variance extracted, and took the average. Hence, both the average variance and correlation were calculated. These scores were used to calculate the two subtypes of construct validity: convergent validity and discriminant validity [51]. It is well documented that for analyse-dimension reduction factor, if the average variance is greater than the correlation then the discriminate validity and convergent validity are established. In the present study, the average variance was more than the correlation and hence both the discriminate and convergent validities were established.

**Fig. 1:**
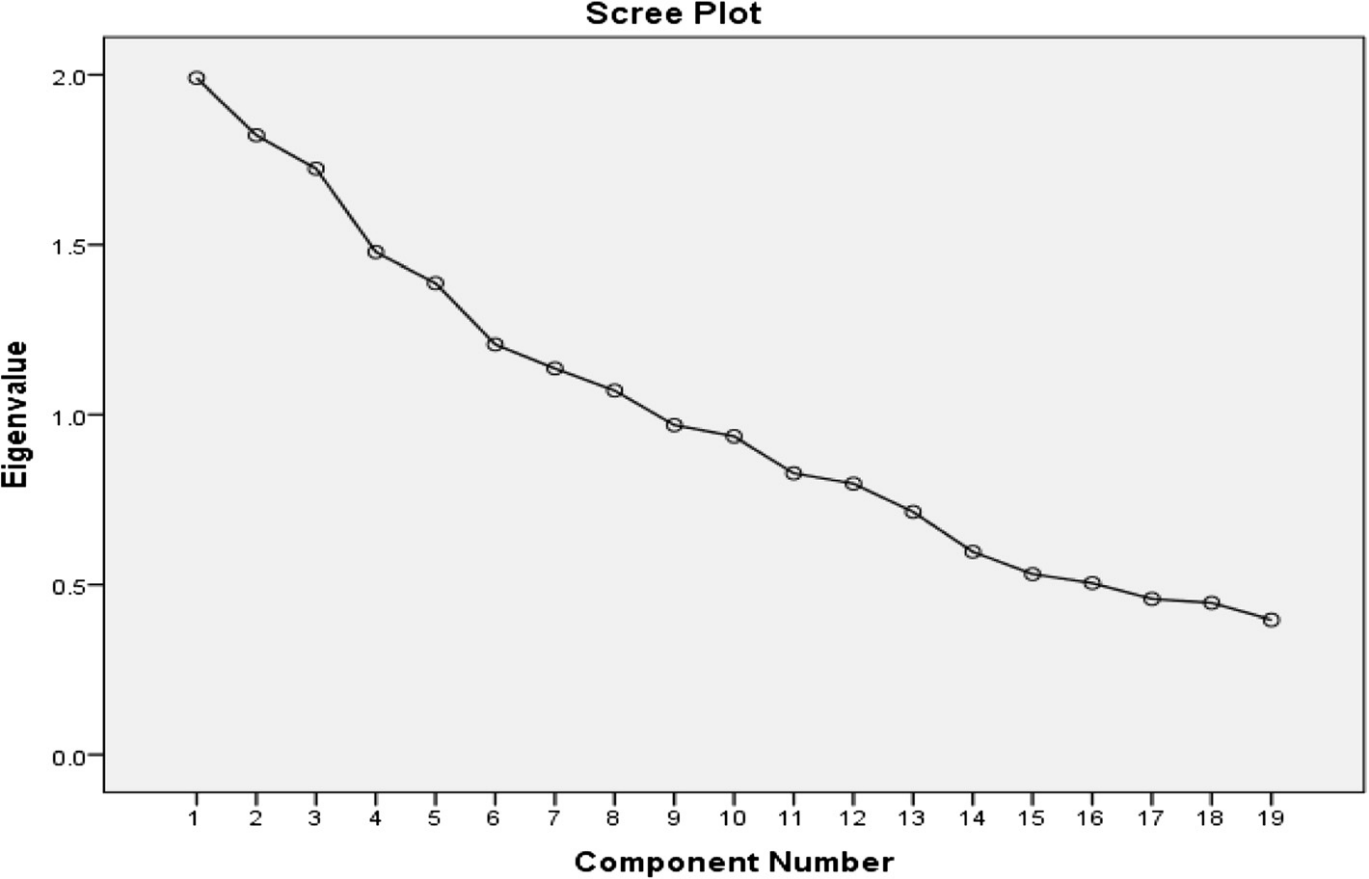
Graphical representation of eigenvalues by scree plot. *X* axis includes the eigenvalues for the motivation components while the *Y* axis represents the different components of motivation

Next, eigenvalues were calculated with each linear component (items/subfactor) before extraction, after extraction and after rotation. It has identified 18 factors within the dataset. The eigenvalues associated with each factor represents the variance explained by that linear component, and it will also display the percentage of variance explained. But it would display only those variance values whose eigenvalues are more than 1 whereas subsequent factors explain only a small amount of variance. The eigenvalues are represented graphically by the scree plot. See Fig. 1 for details.

For understanding of the relative impact of individual factors after extraction, subfactors/items having a values less than 0.6, indicating that variables do not fit well with the factor solution, were dropped from the final analysis (see Table 4 for items included and excluded based on values less that 0.6) and factor analysis was repeated on the remaining 18 items. A revised extraction was further done in the components having a large range of variability within the observations. Observations hence obtained after revised rotation were finally factored into the eight main factors that can be plausible determinants for work-related motivation (see Table 5). The items or subfactors having both positive as well as negative correlation were included as explained in Table 6.

**Table 5:** Rotated component matrix: factors

Factors	Factor 1	Factor 2	Factor 3	Factor 4	Factor 5	Factor 6	Factor 7	Factor 8
Statements/items	Clear duties and responsibilities (statement 4)		Physical safety (statement 25)	Adequate leaves (statement 12)	Pension (statement 10)	Supervisor’s support (statement 19)	Adequate salary and benefits (statement 9)	Growth and development (statement 21)
	Exercising authority (statement 1)	Significant and meaningful goal (statement 2)	Achievement-related promotion (statement 14)	Effective team work (statement 16)	Job meaningfulness (statement 22)	Appreciation for good work (statement 18)	Earned respect as a person (statement 23)	Control over job decision (statement 5)
	Job-related pride and respect (statement 17)		Good working environment (statement 11)					
	Interpersonal relationship (statement 24)							

Extraction method: principal component analysis. Rotation method: varimax with Kaiser normalization

Rotation converged in 24 iterations

**Table 6:** Rotated component matrix: components

	Component							
	1	2	3	4	5	6	7	8
Statement 4	*.714*	−.183				.132		
Statement 1	*.672*	.239	.254	.168	−.120		.196	.151
Statement 17	−.453	.372		.261	.126		−.335	.248
Statement 24	*.441*		−.156	.158	.195	−.200		
Statement 2	−.106	*.765*			.304			
Statement 25			*.658*	−.141				
Statement 14			*.648*	.236		−.171		−.119
Statement 11			*.541*	−.480				.224
Statement 12				*.748*		.223		
Statement 16				−*.700*		.289		
Statement 10		−.130	.138		*.761*		.257	
Statement 22		.169	−.113		*.708*	−.108	−.165	
Statement 19						*.818*		.135
Statement 18	−.187	.176			.104	−*.609*		.388
Statement 9	.126		.117	.115	.201	.104	*.773*	
Statement 23	.183		.371		.269	.106	−*.652*	.142
Statement 21	.294		−.260			−.115	−.145	*.713*
Statement 5	−.327	−.275	.260			.251	137	*.600*

Extraction method: principal component analysis. Rotation method: varimax with Kaiser normalization. Rotation converged in 24 iterations

These eight factors were later labelled and decided upon by developing consensus with authors 1, 2 and 3 and narrated below:

Factor 1: transparency

Factor 2: goals

Factor 3: security (both physical and financial)

Factor 4: convenience

Factor 5: benefits

Factor 6: encouragement

Factor 7: adequacy of earnings

Factor 8: growth and power

Table 6 further explain that of the 18 items, 4 have negative correlation with 8 different components identified.

The rotated component matrix was applied and is narrated in Tables 5 and 6. These 8 factors are narrated according to their positive correlation as explained below:

- The factor of *transparency* included statements 4 and 1 which were positively associated. That means clarity in duties, responsibilities and exercising authority motivate the healthcare workers.
- The factor of *meaningful goals* had a positive value for statement 2 which was a significant and meaningful goal.
- The factor *security* was made up of statements 25, 14 and 11, but statement 11, i.e. good working environment, had a value less than 0.6 and was dropped. It reflects that that a worker is motivated if he/she is provided with basic physical safety (statement 25) and equally important is an achievement-related promotion (statement 14) that possibly keeps the worker motivated.
- The factor of *convenience* did not have any of the positive items other than statement 12 reflecting that adequacy of leaves motivates the staff. However, there were three positive items (adequacy of salary, adequate leaves and physical safety) although less than 0.6 narrating that convenience is one plausible component within motivation that has the potential to motivate the workers.
- The factor of *perceived benefits* was positively correlated with statements 10 and 22 suggesting that pension and job meaningfulness were the most important items that were perceived important by respondents for their motivation.
- The factor *encouragement* had only one statement with a value more than 0.6, and the study participants opined that supervisor’s support (statement 19) is the main item that is related to their motivation.
- The factor *earning* was associated with adequacy of salary and other benefits (statement 9) and hence suggests that salary and other monetary benefits can encourage and motivate the respondents.
- The factor of *growth and power* was associated with growth and development (statement 21) and a control over job decisions related to utilizing money procurement and issues related to HR (statement 5).

## Discussion

The present study was done with an objective to test and develop a reliable and valid instrument for investigating the motivation of health service providers (doctors or MOs and nurses) towards certain job-related aspects and the extent to which these motivate them to perform better at work. Various factors and subfactors were studied and analysed to understand the same. Although the study included 154 respondents, the results cannot be completely generalized to healthcare providers from India and other countries facing similar issues of poor motivation. Further, the study did not assess the current work conditions under which the health workers work, but the results are only based on what healthcare workers perceive about the motivational factors and how much importance they give to different factors for improving their will to perform better at work.

However, despite the limitations, the instrument developed to assess motivation in the current study would be very useful to health reformers, researchers, policy actors and state health systems to design human resource management (HRM) strategies based on motivational needs of healthcare providers that can be assessed using the reliable and valid tool used in the present study. The study is the first of its kind in the country aimed at developing a quantitative tool to assess motivation among public health service providers in India and is based on a solid theoretical framework of motivation [20–23].

Based on literature review, an appropriate instrument containing 19 items was adapted from a study [43]. Next, the instrument was pilot tested and additional seven items were added to the instrument. Pilot testing was done with 14 MOs and 5 nurses working with government health centres from Gujarat (another state), India, during February 2013. These seven additional items were added based on a pilot test of the instrument with health service providers. The modification of the instrument also involved a consensus-developing process among the two experts working on issues of motivation. A two-stage Delphi technique was used to build up the consensus between the experts and the co-authors [44]. The subject expert opinions were also important to develop content validity. The seven additional factors added to the adapted instrument were as follows: job security, availability of adequate resources, physical safety, challenging and interesting work, freely expressing opinion, achievement-related promotion and growth and development. Out of these seven factors, four have been identified as important and have been included in tools used elsewhere [12, 14]. In order to check the tool’s reliability, the Cronbach alpha test statistic was calculated that suggested the Cronbach alpha test statistic value of 0.81 which is an acceptable value for the tool.

To ensure sampling adequacy, the Kaiser-Meyer-Olkin measure of sampling adequacy and Bartlett’s test of sphericity were also applied. Finally, factor analysis was conducted before and after extraction of common variance to calculate the eigenvalues with cutoff values set as 0.6. Based on principal component analysis and after varimax rotation (that was run on the final study instrument with 26 items) with a cutoff value of 0.6, 4 factors that belonged to the original/adapted instrument that contained 19 were excluded. These four factors were as follows: *creative opportunities*, *opportunity to use skill acquired through professional course*, *general decision-making* and *fair treatment by colleagues*. Of these, two are intrinsic factors, i.e. creative opportunities and opportunity to use skill acquired through professional course, while the other two are extrinsic factors.

As far as inclusion and exclusion based on factor analysis results of the seven additional items (that were added to the adapted tool containing 18 factors) was concerned, the item-scale criteria of 0.6 cutoff value did not satisfy in the case of the following four out of seven items: *availability of adequate resources*, *job security*, *freely expressing opinion* and *challenging and interesting work*. While the item-scale criteria of 0.6 cutoff value satisfied for three factors, *achievement-related promotion*, *physical safety* and *growth and development*, indicating the need to include these three items in the final modified version of the tool. Therefore, the final modified tool after running PCA suggested the inclusion of 18 items as follows: exercising authority, significant and meaningful goal, clear duties and responsibilities, control over job decision, adequate salary and benefits, pension, good working environment, adequate leaves, achievement-related promotion, effective teamwork, job-related pride and respect, appreciation for good work, supervisor’s support, growth and development, job meaningfulness, earn respect, interpersonal relationship and physical safety.

Of the final 18 items included in the tool, 7 were intrinsic items, namely the following: *significant and meaningful goal*, *achievement related promotion*, *job-related pride and respect*, *appreciation for good work*, *growth and development*, *job meaningfulness* and *earn respect*, and the remaining 11 were extrinsic factors. According to rotated component matrix, these 18 factors were further labelled under the following 8 main factors: transparency, meaningful goal, security, convenience, perceived benefits, encouragement, earning and growth and power.

Although developing consensus to assess motivation among healthcare providers is subjective, there is an urgent need to develop the tools that can measure the work motivation. Several scales and tools are used in the management studies to ascertain the same, but the present study is the first effort to develop and pilot test the tools in reference to public health system providers in India. The development of the tool in the present study adds a great value to the previous tool and study from Cyprus. First of all, the tool was modified so that the items assessing motivation could be made more explicit to avoid any ambiguity. Secondly, the final tool developed for the study suggested that four factors be excluded from the Cyprus study tool which were as follows: *creative opportunities*, *opportunity to use skill acquired through professional course*, *general decision-making* and *fair treatment by colleagues*. Of these, two are intrinsic factors, i.e. creative opportunities and opportunity to use skill acquired through professional course, while the other two are extrinsic factors. Yet another value addition of the tool developed in the study is that it suggests the need for including *achievement-related promotion*, *physical safety* and *growth and development* indicating a strong need among health service providers towards intrinsic motivation that could potentially have strong policy implications in designing HRH-related strategies that give a strong focus on intrinsic factors of motivation.

It was observed that assessing motivation is a complex phenomenon. But there are certain factors and subfactors that can help in understanding the motivation level of an individual. Observations from the present study indicate that the factors that can assess motivation can be broadly factored into eight domains. Under each domain, there are several subfactors that may motivate an individual. One of the most significant domains that has emerged out of the present tool is physical security that assists in motivating a healthcare provider to be with the system or job.

## Conclusions

In conclusion, this study showed that several motivation factors are important to increase the work motivation of healthcare workers. This study reiterates the fact that intrinsic motivation is an important phenomenon and therefore interventions designed at addressing the motivation must consider intrinsic factors of motivation [10–12, 14]. However, the study findings also indicate that extrinsic factors cannot be ignored as 11 out of 18 items included in the final study tool belonged to extrinsic motivation. Hence, one of the recommendations as supported by research elsewhere is that the state health departments must address the motivation of health service providers by designing a bundle of strategies (a mix of both hygiene and factors of motivation) to respond to the motivational needs of service providers [14, 1]. Therefore, we strongly recommend that the state health departments, policymakers and reformers devise management strategies that address both intrinsic and extrinsic factors of motivation. This study can help in providing researchers and health administrators a tool to assess motivation among healthcare providers, and the results derived from use of the tool can further be useful is designing HRM strategies to address the shortage and maldistribution and improve work performance of health service providers.

This study concludes that motivation factors are important for healthcare workers to improve their will to perform better at work that include both intrinsic and extrinsic motivation factors. Job attribution factor is the highly rated factor among all, and it is an intrinsic factor. It suggests that for health service providers both extrinsic and intrinsic factors are important.

Despite these limitations, this study throws light on some of the motivational factors important for improving healthcare workers’ performance in the healthcare system. There is only little research done on the work motivation of healthcare staff, and this study has the potential to provide health departments and researchers with a tool to assess motivation. However, the authors suggest for a greater need to do research on understanding motivational factors, and in order to do so, there is a need to develop tools with good psychometric properties that can assess motivation.
